# Mycorrhizas alter sucrose and proline metabolism in trifoliate orange exposed to drought stress

**DOI:** 10.1038/srep42389

**Published:** 2017-02-09

**Authors:** Hui-Hui Wu, Ying-Ning Zou, Mohammed Mahabubur Rahman, Qiu-Dan Ni, Qiang-Sheng Wu

**Affiliations:** 1 College of Horticulture and Gardening, Yangtze University, Jingzhou, Hubei 434025, China; 2 Institute of Root Biology, Yangtze University, Jingzhou, Hubei 434025, China; 3 Brix’ N Berries, Leduc, Alberta, Canada; 4 Department of Chemistry, Faculty of Science, University of Hradec Kralove, Hradec Kralove 50003, Czech Republic

## Abstract

Arbuscular mycorrhizal fungi (AMF) can enhance drought tolerance in plants, whereas little is known regarding AMF contribution to sucrose and proline metabolisms under drought stress (DS). In this study, *Funneliformis mosseae* and *Paraglomus occultum* were inoculated into trifoliate orange (*Poncirus trifoliata*) under well watered and DS. Although the 71-days DS notably (*P* < 0.05) inhibited mycorrhizal colonization, AMF seedlings showed significantly (*P* < 0.05) higher plant growth performance and leaf relative water content, regardless of soil water status. AMF inoculation significantly (*P* < 0.05) increased leaf sucrose, glucose and fructose concentration under DS, accompanied with a significant increase of leaf sucrose phosphate synthase, neutral invertase, and net activity of sucrose-metabolized enzymes and a decrease in leaf acid invertase and sucrose synthase activity. AMF inoculation produced no change in leaf ornithine-δ-aminotransferase activity, but significantly (*P* < 0.05) increased leaf proline dehydrogenase activity and significantly (*P* < 0.05) decreased leaf both Δ_1_-pyrroline-5-carboxylate reductase and Δ_1_-pyrroline-5-carboxylate synthetase activity, resulting in lower proline accumulation in AMF plants under DS. Our results therefore suggest that AMF strongly altered leaf sucrose and proline metabolism through regulating sucrose- and proline-metabolized enzyme activities, which is important for osmotic adjustment of the host plant.

Arbuscular mycorrhizal fungi (AMF), a kind of heterotrophic microorganism in soils, can establish a widespread symbiotic association with the roots of ~80% of terrestrial plants, namely, arbuscular mycorrhizas (AMs)^1^,^2^. AMs have the capacity to absorb soil nutrients and water for the host plant, resulting in better plant growth and drought tolerance^1^,^2^,^3^. In return, the host plant provides photosynthates to assist the metabolic activity of AMs^2^,^4^. The mechanisms about AMF-enhanced drought tolerance of host plants are poorly known, though possible mechanisms include direct water and nutrient uptake via extraradical hyphae, better root system architecture, enhancement of antioxidant defense systems, and greater osmotic adjustment^3^,^5^.

Drought stress (DS) is one of the most important abiotic factors unfavorably affecting physiological and biochemical processes in plants^3^,^6^. Osmotic adjustment (OA), a well-known mechanism in response to DS in many plants^7^, refers to a reduction of osmotic potential in response to a net osmolyte accumulation in order to maintain cell turgor for the maintenance of plant metabolic activity and in turn plant growth^8^. In many perennial woody plant species, organic solutes seem to play a main role in OA^7^. In addition, some organic solutes such as glucosylglycerol, 3-dimethylsulphoniopropionate, or β-alanine accumulate only in a few plant species, whereas soluble sugars and proline are widespread in a large number of plants^9^. In osmolytes, sucrose is synthesized in source leaves and constitutes the main carbohydrate form for long-distance transport via symplastic and/or apoplastic pathways to the phloem and then to sink organs such as fruit, roots, and/or AMs^10^. There sucrose is cleaved by either invertase or sucrose synthase (SS, degrading direction)^11^. Invertase, a hydrolase, including acid invertase (AI) and neutral invertase (NI), can cleave sucrose into glucose and fructose, and SS, a glycosyl transferase, converts sucrose into UDP-glucose and fructose in the presence of UDP^12^. In source tissues, sucrose phosphate synthase (SPS) takes part in sucrose synthesis^13^. Earlier studies indicated that in trifoliate orange, AMF inoculation increased AI, NI, and SS activities in leaves but decreased AI, NI, and SS activities in roots^14^. In roots of soybean, mycorrhizal inoculation did not affect AI activities, but increased SS activities 40 days after seed germination^15^. Such changes in sugar accumulation and sucrose-metabolized enzyme activities under mycorrhization highlight AMF’s ability in OA to enhance drought tolerance of host plants.

In addition to sugars, proline, an important osmolyte in plants, plays an important role in the process of OA under DS^16^. Indeed, proline accumulation is likely to a key indicator, which provides plants with an osmotic mechanism to maintain a favourable osmotic potential for water uptake, therefore, alleviating the injury of DS^16^. In plants, proline is synthesized by the glutamate and/or ornithine pathways. The glutamate pathway is the main pathway in proline synthesis, in which glutamate first converts into glutamate-semialdehyde (GSA) by the Δ_1_-pyrroline-5-carboxylate synthetase (P5CS), then spontaneously converts to Δ_1_-pyrroline-5-carboxylate (P5C), and then transforms into proline by Δ_1_-pyrroline-5-carboxylate reductase (P5CR)^16^,^17^,^18^. In the ornithine synthetic pathway, proline can be synthesized from ornithine in mitochondria, where it is first transaminated by ornithine-δ-aminotransferase (OAT), producing GSA and P5C, and then transformed into proline by P5CR^16^,^19^. Proline catabolism occurs in mitochondria, which outlines that proline is first catabolized by proline dehydrogenase (ProDH) producing P5C, and then converted to glutamate by P5C dehydrogenase (P5CDH)^16^. AMF inoculation often induces proline accumulation^17^,^19^,^20^ or proline decrease^5^,^21^,^22^ in drought-stressed plants. Wu *et al*.^5^ concluded that AMF seedlings accumulated less proline than non-AMF seedlings in leaves of citrus exposed to DS, due to either greater drought resistance or less injury in AMF seedlings under DS. Similar findings were reported for other species, such as *Antirhinum majus*^3^, *Erythrina variegate*^6^, *Cyclobalanopsis glauca*^21^ and *Ocimum gratissimum*^22^. In contrast, higher proline level in AMF plants exposed to DS was also found in *Oryza sativa*^17^ and *Macadamia tetraphylla*^19^. However, the effect of AMF on proline metabolism is still not fully understood.

Citrus, one of the most important fruit crops grown in many regions of the world, is highly sensitive to soil drought stress (DS). Moreover, citrus plants are strongly dependent on the AM symbiosis^4^. Trifoliate orange (*Poncirus trifoliata* L. Raf.), a close relative to *Citrus*, is widely used as the main rootstock of citrus plantations in Asia, including China, India, and Japan. Studies confirm that AMF inoculation enhanced drought tolerance in citrus plants^5^,^23^,^24^,^25^. Nevertheless, little is known regarding the contribution of AMF to sucrose- and proline-metabolized enzyme activities in trifoliate orange exposed to DS, despite that the changes of sugars and proline concentration induced by AMF have been reported^5^. In this background, the objectives of the present study are to elucidate the effects of AMF on organic solute contents in trifoliate orange subjected to well watered (WW) and DS conditions and to analyze the changes in sucrose- and proline-metabolized enzyme activities.

## Results

### Root AMF colonization

Mycorrhizal colonization was not observed in roots of non-AMF seedlings regardless of WW and DS. Root mycorrhizal colonization in inoculated plants varied from 34.2% to 57.5% with *F. mosseae* and 49.7% to 70.5% with *P. occultum*, respectively (Table 1). DS treatment significantly (*P* < 0.05) decreased root colonization by *F. mosseae* and *P. occultum*. In addition, mycorrhizal colonization by *P. occultum* was markedly (*P* < 0.05) higher than by *F. mosseae*, regardless of soil water status. There was a significant interaction between soil water treatments and AMF treatments (*P* < 0.01) (Table 1).

### Plant growth performance

Compared with non-AMF treatment, *F. mosseae* and *P. occultum* treatments significantly (*P* < 0.05) increased plant height, stem diameter, leaf number, and leaf, stem, and root dry weight under both WW and DS, whilst inoculation with *P. occultum* showed better effects on stimulating plant growth than with *F. mosseae* (Table 1).

### Leaf relative water content (RWC)

**Figure 1:**
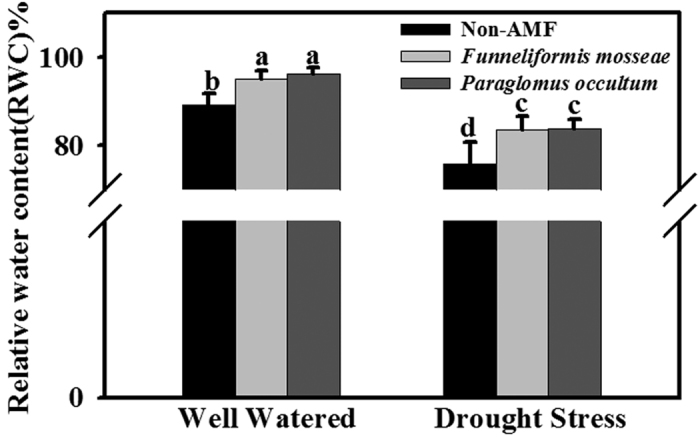
Effect of *Funneliformis mosseae* and *Paraglomus occultum* on leaf relative water content (RWC) of trifoliate orange seedlings under well watered (WW) and drought stress (DS) conditions. Data (mean ± SD, *n* = 5) followed by different letters above the bars among treatments indicate significant differences at 5% level.

AMF seedlings by *F. mosseae* and *P. occultum* showed significantly (*P* < 0.05) higher RWC than non-AMF seedlings under WW and DS conditions, respectively (Fig. 1). No significant interaction between water treatments and AMF treatments was observed (Table 2).

### Leaf carbohydrate concentrations

**Figure 2:**
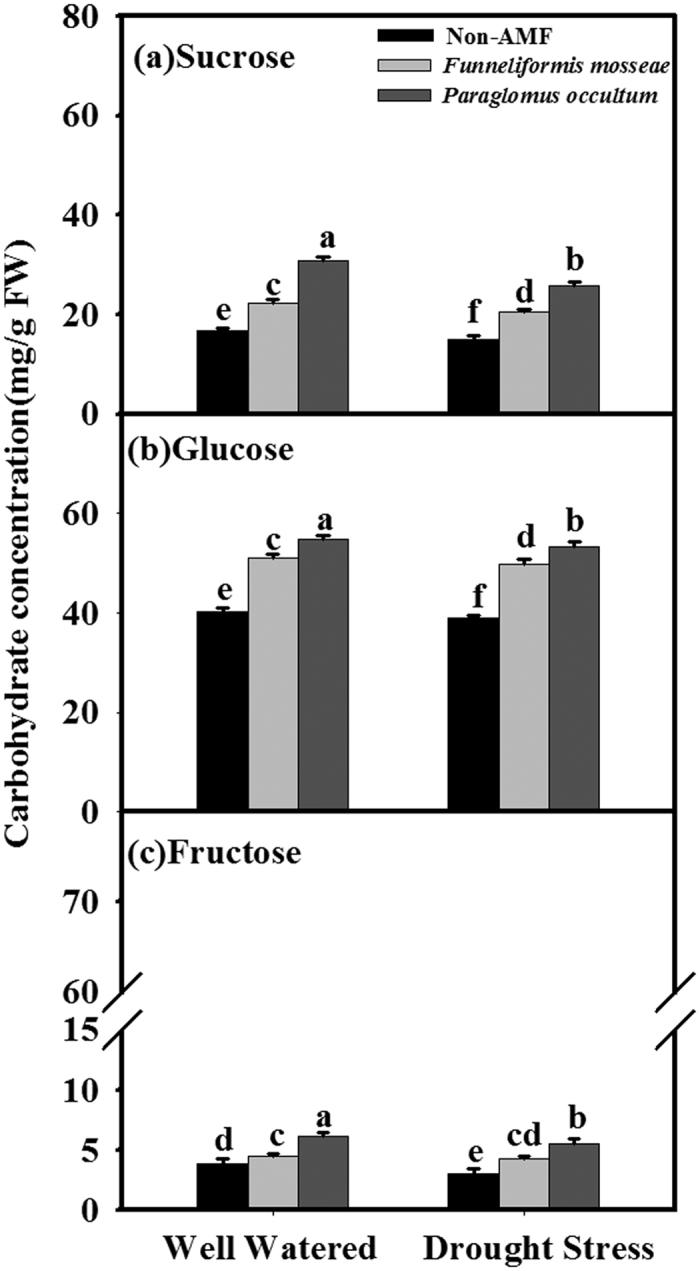
Effects of *Funneliformis mosseae* and *Paraglomus occultum* on carbohydrate concentrations in leaves of trifoliate orange seedlings under well watered (WW) and drought stress (DS) conditions. Data (mean ± SD, *n* = 5) followed by different letters above the bars among treatments indicate significant differences at 5% level.

The seedlings colonized by *P. occultum* and *F. mosseae* recorded significantly (*P* < 0.05) higher leaf fructose, glucose, and sucrose concentrations than non-AMF colonized seedlings, regardless of WW or DS (Fig. 2a–c; Table 2). Significantly higher fructose, glucose, and sucrose levels were found in leaves of AMF seedlings inoculated with *P. occultum* compared with those inoculated with *F. mosseae*, irrespective of soil water status (Fig. 2a–c).

### Activities of sucrose-metabolized enzymes in leaves

**Figure 3:**
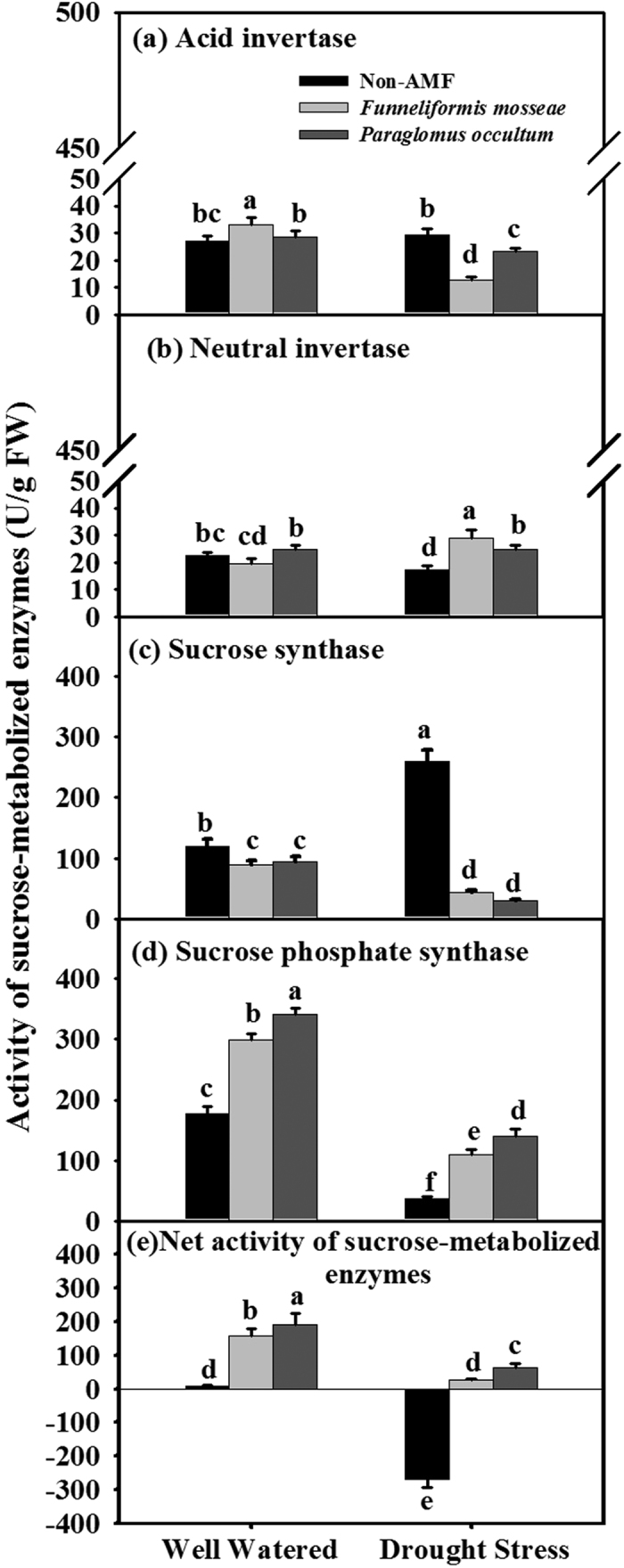
Effects of *Funneliformis mosseae* and *Paraglomus occultum* on activities of sucrose-metabolized enzymes in leaves of trifoliate orange seedlings under well watered (WW) and drought stress (DS) conditions. Data (mean ± SD, *n* = 5) followed by different letters above the bars among treatments indicate significant differences at 5% level.

Under WW conditions, treatment with *F. mosseae* significantly (*P* < 0.05) increased leaf AI and SPS activity by 23.5% and 69.2%, and inoculation with *P. occultum* increased leaf SPS activity by 92.5%, compared with non-AMF treatment (Fig. 3a–d). AMF seedlings colonized by *F. mosseae* and *P. occultum* also showed 25.6% and 20.7% significantly (*P* < 0.05) lower leaf SS activity than non-AMF seedlings under WW condition. Under DS condition, mycorrhization with *F. mosseae* and *P. occultum* markedly (*P* < 0.05) increased leaf NI activity by 69.2% and 45.8% and leaf SPS by 198.1% and 282.0% respectively. They also decreased leaf AI by 57.0% and 20.2% and leaf SS activity by 83.5% and 88.5%, respectively (Fig. 3a–d). AMF seedlings colonized by *F. mosseae* and *P. occultum* showed notably (*P* < 0.05) higher net activity of sucrose-metabolized enzymes regardless of soil water status (Fig. 3e). There was the significant interaction in leaf AI, NI, SPS, and net activity of sucrose-metabolized enzymes between soil water condition and AMF treatment (*P* < 0.01) (Table 2).

### Proline concentration

**Figure 4:**
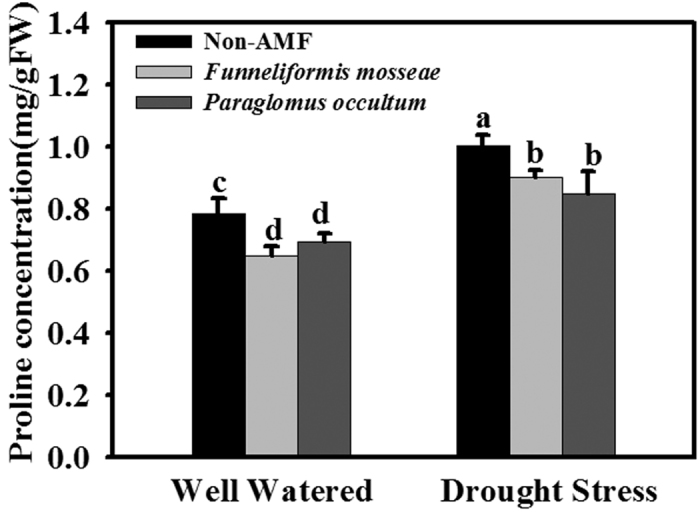
Effects of *Funneliformis mosseae* and *Paraglomus occultum* on proline concentration in leaves of trifoliate orange seedlings under well watered (WW) and drought stress (DS) conditions. Data (mean ± SD, *n* = 5) followed by different letters above the bars among treatments indicate significant differences at 5% level.

AMF seedlings colonized by *F. mosseae* and *P. occultum* showed significantly (*P* < 0.05) lower leaf proline level under WW and DS conditions (Fig. 4). There was no significant difference in leaf proline concentration between *F. mosseae* and *P. occultum* (Fig. 4) and a significant (*P* < 0.05) interaction between soil water treatments and AMF treatments (Table 2).

### Activities of proline-metabolized enzymes in leaves

**Figure 5:**
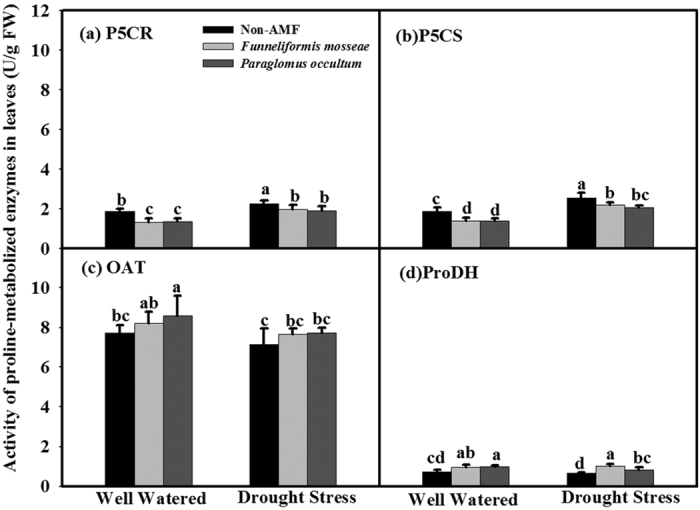
Effects of *Funneliformis mosseae* and *Paraglomus occultum* on activity of proline-metabolized enzymes in leaves of trifoliate orange seedlings under well watered (WW) and drought stress (DS) conditions. Data (mean ± SD, *n* = 5) followed by different letters above the bars among treatments indicate significant differences at 5% level.

In gereral, *F. mosseae*- and *P. occultum*-treated seedlings showed significantly (*P* < 0.05) lower leaf P5CS and P5CR activity than non-AMF-treated seedlings under WW and DS activity (Fig. 5a,b; Table 2). Only *P. occultum* treatment significantly (*P* < 0.05) increased leaf OAT activity compared with non-AMF treatment under WW condition (Fig. 5c; Table 2). Treatment with *F. mosseae* and *P. occultum* significantly (*P* < 0.05) increased leaf ProDH activity compared to non-AMF treatment under both WW and DS (Fig. 5d; Table 2). A significant (*P* < 0.05) interaction between soil water treatments and AMF treatments was observed for ProDH activity (Table 2).

### Correlation studies

Under WW condition, leaf SS activity was significantly (*P* < 0.05) negatively correlated with leaf glucose concentration, and activity of leaf SPS and net activity of sucrose-metabolized enzymes were significantly (*P* < 0.01) and positively correlated with leaf fructose, glucose, and sucrose concentrations (Table 3). Under DS condition, leaf AI activity was significantly (*P* < 0.05) negatively correlated with leaf glucose concentration, and leaf SS activity was significantly (*P* < 0.01) and negatively correlated with leaf fructose, glucose, and sucrose concentration. Activity of leaf NI, SPS and net activity of sucrose-metabolized enzymes were significantly (*P* < 0.05 or *P* < 0.01) and positively correlated with leaf fructose, glucose, and sucrose concentrations (Table 3).

Under WW conditions, leaf proline concentration was significantly (*P* < 0.01) positively correlated with leaf P5CR and P5CS activity (Table 3). Under DS condition, leaf proline concentration was significantly (*P* < 0.05) positively correlated with leaf P5CR activity and negatively with leaf ProDH activity (Table 3).

## Discussion

### Mycorrhizal roles in plant growth under drought stress

This study showed a significant (*P* < 0.05) decrease in root AMF colonization by DS. Similar results had been reported in other plant species, such as sweet potato^26^, *Cyclobalanopsis glauca*^21^ and *Cucumis melo*^27^. The negative effects of drought stress on root AMF colonization might be due to the germination of spores and the spread of hyphae in soils being inhibited by DS^21^,^27^.

Although the decrease of root colonization under DS was represented, such AMF colonization still significantly promoted plant growth parameters of trifoliate orange grown under DS conditions. This result is consistent with previous works using *Erythrina variegate*^6^ and *Poincianella pyramidalis*^28^. Enhancement of growth and biomass and higher leaf RWC in AM plants could be due to improved water and nutrient uptake assisted by mycorrhizal hyphae^3^,^6^,^28^. In addition, *P. occultum* had markedly (*P* < 0.05) greater plant growth performance than *F. mosseae* under DS, which is closely related with considerably higher root mycorrhizal colonization under *P. occultum* than *F. mosseae*. It also suggests that growth improvement of trifoliate orange by AMF is strongly dependent on AMF species.

### Mycorrhizal roles in sucrose metabolism under drought stress

Carbohydrates are considered as the important compatible solutes for OA in plants under DS^7^. In the present study, AMF colonization by *F. mosseae* and *P. occultum* significantly increased leaf sucrose, fructose, and glucose concentrations under DS conditions, which would act as osmolytes to protect and stabilize plant macromolecules and structures from drought damage, thereby enhancing the drought tolerance of the host plant by OA^9^. A relatively higher RWC was observed in AM trifoliate orange seedlings than in non-AM seedlings under DS, further suggesting greater water status in AM plants subjected to DS. These results are in agreement with earlier studies^3^.

In this study, leaf SPS activity was significantly higher in AMF seedlings than in non-AMF seedlings exposed to WW and DS. This is in agreement with the findings of Zhu *et al*.^29^, who reported a higher SPS activity in *G. tortuosum*-colonized *Zea mays* under low temperature (15 °C for 2 weeks). Moreover, our results further indicated that leaf SPS activity was significantly (*P* < 0.01) and positively correlated with leaf fructose, glucose, and sucrose concentrations under DS conditions. Higher leaf carbohydrate accumulation in AMF seedlings was caused by an AMF-induced increase in leaf SPS activity. This shows that AM symbiosis can modulate SPS activity to induce sugar accumulation.

In general, sucrose needs to be cleaved by sucrose-cleaving enzymes (AI, NI, and SS) into glucose and fructose, while glucose can be absorbed directly by mycorrhizal formations^30^. The present study showed that leaf AI activity under WW was increased by *F. mosseae* but was reduced by *F. mosseae* and *P. occultum* under DS. Similarly, root colonization by *F. mosseae* and *P. occultum* did not alter leaf NI activity under WW, but significantly increased leaf NI activity under DS. These results indicated that soil water strongly affects the behavior of AMF on leaf AI and NI activity. Significantly lower leaf SS activity was found in AMF seedlings than in non-AMF seedlings under DS, irrespective of *F. mosseae* or *P. occultum*. Similar results of the AMF-induced SS changes were also found in *Citrus tangerina* colonized by *F. mosseae*^4^ and trifoliate orange colonized by *F. mosseae* grown in 3 mM P level of the growth substrate^31^.

Sugar accumulation is relative to dynamic balance between the activity of sucrose synthetic enzymes (SPS) and sucrose-cleaving enzymes (AI, NI and SS), and thus net activity of sucrose-metabolized enzymes plays a dominant role in sugar accumulation^13^. In this study, AMF inoculation with *F. mosseae* and *P. occultum* strongly stimulated an increase in net activity of sucrose-metabolized enzymes, regardless of WW or DS. Net activity of sucrose-metabolized enzymes was significantly and positively correlated with sucrose, glucose, and fructose concentration in leaves. This result suggested that AMF-induced sugar accumulation might be associated with an increase in the net activity of sucrose-metabolizing enzymes. It seems that AM symbiosis might modulate net activity of sucrose-metabolized enzymes to induce sugar accumulation, which is beneficial to OA. AMF-induced sucrose-metabolized enzyme changes will need to be studied by analyzing their gene regulation through qRT-PCR.

### Mycorrhizal roles in proline metabolism under drought stress

Data from the present study showed a lower accumulation of proline in leaves from AMF trifoliate orange plants than non-AMF plants under DS. This result is in agreement with previous findings in soybean^32^, *Erythrina variegate* plants^6^ and *Ocimum gratissimum*^24^ under DS. As stated by Augé and Moore^33^, lower accumulation of proline caused by mycorrhization may be due to less strain by DS, because of greater water status in AMF plants. The present work revealed drought stress induced higher proline concentrations accompanied by an increase of P5CR and P5CS activity, a decrease of OAT activity and no difference of ProDH in leaves of trifoliate orange, suggesting that proline accumulation in AMF and non-AMF trifoliate orange was derived from the enhancement of the glutamate synthetic pathway of proline but not the ornithine synthetic pathway of proline. This is in accordance with previous studies of Zou *et al*.^34^. In this study, AMF seedlings showed significantly lower P5CR and P5CS activity but substantially higher ProDH activity than non-AMF seedlings, irrespective of WW or DS conditions. This means that a decrease in proline accumulation in AMF seedlings is potentially associated with an AMF-modulated decrease of glutamate synthetic pathways and an increase of proline catabolism, which will be still studied by checking the relevant gene expression.

In short, AMF seedlings showed significantly higher leaf fructose, glucose, and sucrose concentrations and lower leaf proline accumulation, due to the regulation of sucrose- and proline-metabolized enzyme activities by mycorrhization, which is beneficial to OA in the host plant. Nevertheless, the underlying molecular mechanisms in AMF plants still need to be further examined by RNA-seq.

## Methods

### Experimental set-up

The seeds of trifoliate orange were surface-disinfected in a 70% ethanol solution for 15 minutes, subsequently rinsed with distilled water, and then germinated in autoclaved (121 °C, 0.11 MPa, 2 h) river sand in a controlled growth microcosm at 28/20 °C and 16/8 photoperiod hours (day/night) with 80% relative humidity and 1200 μmol/m^2^/s photon flux density. After three weeks, two 4-leaf-old seedlings with the same size were transplanted into a plastic pot (11.8 cm upper diameter × 8.9 cm bottom diameter × 14 cm height) filled with autoclaved growth substrate (soil/sand = 4/1, v/v). The soil was collected from a citrus orchard of the Yangtze University campus and taxonomically classified as Xanthi-Udic Ferralsols (FAO system) and the sand came from Yangtze River. The physio-chemical characteristics of the growth substrate are: pH of 6.2, 8.5 g/kg organic carbon, 10.3 mg/kg available nitrogen, and 13.3 mg/kg Oslen-P.

Two AMF species, *Funneliformis mosseae* (Nicol. & Gerd.) Schüßler & Walker and *Paraglomus occultum* Walker Morton and Redecker, were used here. These AMF species were purchased from the Bank of Glomeromycota in China and were propagated through identified spores with *Trifolium repens* for 16 weeks. Approximately 1000 spores/pot of each AM fungus were applied at transplanting of the seedlings. Non-AMF treatment also received the equivalent quantity sterilized inocula and 2 mL inocula filtrate (25 μm filter) to keep similar microbial communities except the AM fungus. All the seedlings were grown in a glass greenhouse with the characteristics of photosynthetic photon flux density of 728‒965 μmol/m^2^/s, day/night temperature 20–35/15–26 °C, and relative humidity 70–95%.

### Water treatments

Water treatments began 87 days after seedling transplanting. Half of the seedlings were subjected to WW by gravimetrically maintaining 70% of the maximum water holding capacity of the substrate for 71 days. The other seedlings were exposed to DS via maintaining 50% of the maximum water holding capacity of the substrate for 71 days. Water status in each pot was maintained daily by weighing and any loss of water was resupplied to maintain the target soil relative water content. The location of pots was shifted weekly to avoid environmental differences.

### Experimental design

The experiment was designed using a 3 × 2 randomized complete block design with three inoculations with AMF (*F. mosseae, P. occultum,* and non-AMF) and two soil water regimes (WW and DS). Each treatment was replicated five times, requiring a total of 30 pots for this study.

### Variable determinations

After 71 days of water treatments, the seedlings were harvested and growth parameters such as plant height, stem diameter, and leaf number per plant were recorded. The seedlings were divided into shoots and roots and the dry weight of each was measured after baking at 75 °C for 48 h.

A portion of fresh roots were cut into 1-cm long root segments, cleared in 10% (w/v) KOH at 90 °C for 1.5 h, acidified in 20 mM HCl for 10 min, and finally stained with 0.05% trypan blue in lactophenol^35^. Root AMF colonization was expressed as the percentage of infected root lengths by AMF against observed total root lengths.

Leaf relative water content (RWC) was assessed by the method employed by Huang *et al*.^24^.

Fructose, glucose and sucrose concentration in leaves was determined by the protocol outlined by Wu *et al*.^31^.

A 0.2-g fresh leaf sample was homogenized in a chilled mortar with 4 mL 100 mM Hepes–NaOH buffers (pH 7.5), containing 20 mM EDTA, 1 mM NaF, 1 mM benzamidine, 20 mM cysteine and 1% polyvinyl pyrrolidone. The mixture was centrifuged at 10,000 × g for 30 min, and the supernatants were dialyzed with 100 mM Hepes–NaOH buffer (pH 7.5) in a 21-mm dialysis bag for 12 h at 4 °C. The activity of AI and NI was determined by the protocol described by Wu *et al*.^4^. To determine SPS activity, 40 μL dialyzed supernatant was added into 100 μL reaction mixtures including 5 mM fructose-6-phophate, 10 mM uridine diphosphate glucose, 15 mM MgCl_2_, 15 mM glucose-6-phophate, 1 mM EDTA, and 50 mM Hepes-KOH buffer (pH 7.5). The 140 μL mixtures were incubated at 30 °C for 30 min, terminated with 0.2 mL 5 mM NaOH at 100 °C for 10 min, mixed with 3.5 mL anthrone solution (0.15 g anthrone + 100 mL 81% H_2_SO_4_) at 40 °C for 20 min, and then measured according to Hubbard *et al*.^15^. The activity of SS (degradative direction) was determined according to Lowell *et al*.^36^. Mixtures containing 80 mM Mes buffers (pH = 5.5), 5 mM NaF, 100 mM sucrose and 5 mM UDP were incubated at 30 °C for 30 min, terminated with DNS at 100 °C for 5 min, and the absorbance of the mixtures was recorded at 540 nm. Net activity of sucrose-metabolized enzymes was calculated according of Hubbard *et al*.^13^ with the following formula: net activity of sucrose-metabolized enzymes = SPS activity − (AI + NI + SS) activity.

Proline concentration in leaves was determined using the ninhydrin method of Troll and Lindsley^37^. The activity of leaf P5CS, OAT, and ProDH was assayed according to Zou *et al*.^34^. The activity of leaf P5CR was determined according to the method of Chilson *et al*.^38^ with minor modifications. Briefly, 0.2 g of fresh leaf samples was homogenized with 5 mL 100 mM Tris-HCl buffer (pH 7.5) containing 10 mM MgCl_2_, 1 mM EDTA, 10 mM β-mercaptoethanol, 2 mM phenylmethanesulfonyl fluoride and 2% (w/v) polyvinylpolypyrrolidone, and then centrifuged at 20,000 × g for 20 min at 4 °C. The enzyme activity was assayed with a final volume of 1.0 mL reaction mixture containing supernatant, 200 mM glycine buffer (pH 10.3), 15 mM NAD^+^, and 20 mM proline. The absorbance at 340 nm was recorded and one unit of P5CR was defined as the enzyme amount of 1 μmol NADH during 1 min (U/g FW).

### Statistical analysis

Data (means ± SD, *n* = 5) was performed using the two-way analysis of variance (ANOVA) with SAS software (8.1 v, SAS Institute Inc., Cary, NC, USA), and the significant differences between the treatments were compared with the Duncan’s multiple range test at *P* < 0.05. Pearson correlation coefficients between variables were tested by the CORR procedure based on SAS software.

## Additional Information

**How to cite this article**: Wu, H.-H. *et al*. Mycorrhizas alter sucrose and proline metabolism in trifoliate orange exposed to drought stress. *Sci. Rep.* **7**, 42389; doi: 10.1038/srep42389 (2017).

**Publisher's note:** Springer Nature remains neutral with regard to jurisdictional claims in published maps and institutional affiliations.

## Figures and Tables

**Table 1:** Effects of *Funneliformis mosseae* and *Paraglomus occultum* on root AMF colonization and growth performance of trifoliate orange seedlings under WW and DS.

Water status	AMF status	Root AMF colonization (%)	Plant height (cm)	Stem diameter (mm)	Leaf number	Biomass (g DW/plant)		
						Leaf	Stem	Root
WW	+*F. mosseae*	57.5 ± 5.5b	43.0 ± 2.2b	3.58 ± 0.14b	33.3 ± 2.0b	0.42 ± 0.02b	1.12 ± 0.05b	0.93 ± 0.07b
	+*P. occultum*	70.5 ± 5.6a	59.1 ± 1.8a	3.92 ± 0.19a	42.8 ± 2.8a	0.73 ± 0.05a	1.67 ± 0.06a	1.37 ± 0.11a
	Non-AMF	0.0 ± 0.0e	25.3 ± 1.6e	2.59 ± 0.07d	24.1 ± 1.2c	0.26 ± 0.02d	0.53 ± 0.02e	0.63 ± 0.03d
DS	+*F. mosseae*	34.2 ± 3.0d	29.7 ± 2.4d	2.72 ± 0.17d	23.0 ± 1.6c	0.26 ± 0.03d	0.59 ± 0.05d	0.65 ± 0.10 cd
	+*P. occultum*	49.7 ± 5.1c	39.2 ± 2.5c	2.98 ± 0.18c	33.6 ± 2.9b	0.32 ± 0.03c	0.82 ± 0.05c	0.75 ± 0.05c
	Non-AMF	0.0 ± 0.0e	20.1 ± 1.2f	2.28 ± 0.08e	19.3 ± 0.8d	0.21 ± 0.02e	0.46 ± 0.01f	0.52 ± 0.04e
*Signification*								
AMF		**	**	**	**	**	**	**
DS		**	**	**	**	**	**	**
AMF × DS		**	**	**	*	**	**	**

Data (mean ± SD, *n* = 5) followed by different letters among treatments indicate significant differences at 5% level. *P < 0.05; **P < 0.01; NS: not significant.

Abbreviation: AMF, arbuscular mycorrhizal fungi; DS, drought stress; WW, well watered.

**Table 2:** Significance of variable variations between AMF and non-AMF colonized seedlings under WW and DS.

	AMF	DS	AMF × DS
Fructose concentration	**	**	NS
Glucose concentration	**	**	NS
Sucrose concentration	**	**	**
AI	**	**	**
NI	**	NS	**
SS	**	NS	**
SPS	**	**	**
Net activity of sucrose-metabolized enzymes	**	**	**
RWC	**	**	NS
Proline concentration	**	**	*
P5CR	**	**	NS
P5CS	**	**	NS
OAT	*	**	NS
ProDH	**	NS	*

*P < 0.05; **P < 0.01; NS: not significant. Abbreviation: AI, acid invertase; AMF, arbuscular mycorrhizal fungus; DS, drought stress; NI, neutral invertase; OAT, ornithine-δ-aminotransferase; P5CR, Δ_1_-pyrroline-5-carboxylate reductase; P5CS, Δ_1-_pyrroline-5-carboxylate synthetase; ProDH, proline dehydrogenase; RWC, relative water content; SPS, sucrose phosphate synthase; SS, sucrose synthase (degrading direction); WW, well watered.

**Table 3:** Pearson correlation coefficients (*n* = 15) between activity of sucrose-metabolized enzymes and carbohydrate concentrations in leaves or between activity of proline-metabolized enzymes and proline concentrations in leaves of trifoliate orange seedlings.

	AI			NI			SS			SPS			Net activity of sucrose-metabolized enzymes			Proline			
	Fructose	Glucose	Sucrose	Fructose	Glucose	Sucrose	Fructose	Glucose	Sucrose	Fructose	Glucose	Sucrose	Fructose	Glucose	Sucrose	P5CR	PSCS	OAT	ProDH
WW	−0.03	0.28	0.09	0.39	0.14	0.40	−0.25	−0.60*	−0.40	0.81**	0.98**	0.91**	0.74**	0.97**	0.86**	0.65**	0.82**	−0.35	−0.50
DS	−0.35	−0.57*	−0.34	0.60*	0.77**	0.61*	−0.84**	−0.97**	−0.88**	0.92**	0.98**	0.95**	0.87**	0.98**	0.90**	0.62*	0.48	−0.41	−0.52*

^*^P < 0.05; **P < 0.01. Abbreviation: AI, acid invertase; DS, drought stress; NI, neutral invertase; OAT, ornithine-δ-aminotransferase; P5CR, Δ_1_-pyrroline-5-carboxylate reductase; P5CS, Δ_1-_pyrroline-5-carboxylate synthetase; ProDH, proline dehydrogenase; SPS, sucrose phosphate synthase; SS, sucrose synthase; WW, well watered.
